# Towards a clinical pathway to food for special medical purpose in China: current progress based on a cross-sectional survey

**DOI:** 10.3389/fmed.2025.1538105

**Published:** 2025-07-04

**Authors:** Jie Gong, Mengyan Wang, Jiankui Guo, Yishu Lyu, Dongyu Mu, Lei Shi, Wen Hu, Fengmei Yu

**Affiliations:** Department of Clinical Nutrition, West China Hospital, Sichuan University, Chengdu, China

**Keywords:** food for special medical purpose, survey, clinical pathway, tertiary medical institutions, China, food regulation

## Abstract

**Background and objectives:**

To investigate the current status of clinical pathway implementation for food for special medical purpose (FSMP) in China and provide a scientific basis for constructing a standardized pathway.

**Methods and study design:**

An E-questionnaire was distributed to 27 clinical nutrition quality control centers in tertiary medical institutions in China from September to October 2023 via random stratified sampling.

**Results:**

Ninety-eight valid questionnaires were ultimately recovered. The number of FSMPs used ranged from 1 to 33. The rates of active nutritional risk screening, nutritional status assessment and diagnosis before FSMP therapy were 93.87, 93.88, and 97.96%, respectively. In addition to nutritional physicians, dietitians, and clinicians, nurses participated in prescribing FSMP in 12.24% of the hospitals. Before a prescription was issued, 65 (66.33%) hospitals had an audit process conducted by superior clinicians or dietitians. The frequency of routine ward rounds for more than half of the hospitalized inpatients was once a day. Post-discharge follow-up was implemented in 57 (58.16%) hospitals. The preparation of FSMP in 77 (87.50%) hospitals was included in the supervision of nosocomial infection. The frequency of infection supervision in half of the hospitals was once a month. Sixty-four (65.31%) hospitals had established monitoring and treatment plans for FSMP adverse reactions. Eighteen (18.37%) hospitals had set up FSMP counters for patients. Outpatients from 79 (80.61%) hospitals received FSMP in the department of clinical nutrition. Forty-five (45.92%) hospitals had charge codes. More than 20 different types of charges were collected.

**Conclusion:**

An FSMP clinical pathway prototype (Nutritional Screening-Assessment-Diagnosis-Treatment) has been implemented in China’s tertiary medical institutions. However, many irregularities exist. A standardized clinical pathway with universality and enforceability needs to be developed and promoted. There is an urgent need for China to strengthen its regulation policies and for other countries to share their experiences in the clinical application of FSMP.

## 1 Introduction

In China, food for special medical purpose (FSMP) refers to specially processed and formulated food formulas used to meet the special nutritional or dietary needs of people with limited intake, impaired digestion and absorption, and metabolic disorders or specific disease states (1). FSMP has long been commonly used in developed countries such as the United States (US), Canada, and Australia. In 2013, China officially clarified its definition and categorization. Although the names are not exactly the same in different countries, such as “Medical Foods” in the US and “Foods for Special Dietary Use” in Canada (2–4), the definitions and uses of FSMP are basically the same. The products are positioned as a kind of special food different from ordinary food and drugs, and most of the definitions in various countries reflect that these foods are intended to meet the special needs of patients with limited or impaired ability to eat, digest, and absorb nutrients or diets, and require the use of the foods under the guidance of physicians or clinical dietitians (3–6).

Medical nutrition therapy has a long history in China. The culture of Chinese food therapy dates back thousands of years. It remains deeply cherished and widely practiced by the general public. The concept of using food for disease prevention and treatment has gained broad public acceptance. Therefore, when FSMPs entered the Chinese market, they experienced rapid industry growth. China has given unprecedented protection to all aspects of FSMP predevelopment, clinical trials, registration, production, and distribution. China has far more relevant standards, management methods, and supporting documents than other countries, which may be related to China’s current stage of change in adapting to the rapid growth of such foods.

With the increased clinical practice of FSMP, problems have been identified in the management and clinical application of FSMPs in Chinese hospitals. Many problems exist in the management and clinical application of FSMPs in Chinese hospitals. Actually, few medical institutions have established FSMP management committees (7). The Sichuan Clinical Nutrition Quality Control Center has received 148 reports of FSMP adverse reactions (ADRs) since the establishment of an ADR monitoring system in 2017, and more than 60% of these cases were due to irrational use in the clinic (8). Furthermore, underapplications and overapplications coexist. A survey of the application of nutritional support to inpatients in China revealed that only 32.8% of the patients with nutritional risk received nutritional support, and 10% of patients without nutritional risk received nutritional support (9).

Current research is expanding its focus to the development of FSMP products and their effectiveness in the treatment of disease (10–19). However, whether a high-quality FSMP product can play a proper role in nutritional therapy depends more on whether it is managed in a standardized manner and used reasonably. The process of nutritional screening-nutritional assessment-intervention-adverse effect monitoring is used in intervention studies (20). However, in clinical practice, the actual process is much more complex. The order of the process and the person who performing the process have an important impact on FSMP treatment. In this regard, no references to international experience with clinical pathway regulation were found.

A initial clinical pathway for FSMP was proposed in 2021 by a tertiary care pediatric hospital in China based on its 10-years of experience in the use of various enteral nutrition preparations in pediatrics: The doctor will issue a FSMP prescription, the nurse will check the prescription, and then the dietitian will be responsible for reviewing it, as well as preparing and distributing (21). Nurses are responsible for keeping the special food after receiving it and distributing it to patients. In the same year, the Expert Consensus on Clinical Management of FSMP was released, which further guided the clinical application of FSMP in China and provided professional advice on certain links of clinical application. In 2023, a comprehensive evaluation index system for the rational use of FSMP in medical institutions was proposed to standardize the management of FSMP as well as promote the rational use of FSMP. However, no standardized clinical pathways have been proposed and promoted so far.

Standardized FSMP clinical pathway management is conducive to promoting the quality of clinical nutrition practice. Clarifying the current status of FSMP clinical pathways in Chinese medical institutions is the prerequisite and foundation for developing scientific clinical pathways. This would also contribute to the healthy development of the FSMP industry. Therefore, this study focused on China’s tertiary medical institutions, investigated the current status of FSMP clinical pathway implementation, and provided a scientific basis for constructing a standardized pathway for the clinical use of FSMPs, which could also serve as a valuable regulatory reference for other nations.

## 2 Materials and methods

### 2.1 Study design

A cross-sectional, nationwide, multicenter survey was conducted from September 2023 to October 2023 in China. An E-questionnaire was distributed to tertiary care institutions in China (except Hong Kong, Macao, and Taiwan) using computerized random stratified sampling.

### 2.2 Questionnaire design

A questionnaire entitled “Survey on the Current Situation of Pathways for the Clinical Use of FSMP in Medical Institutions in the Nation” (Supplementary material S1) was formulated based on the comprehensive evaluation index system for the rational use of FSMP in medical institutions and the Expert Consensus on Clinical Management of FSMP (2021 Edition (22, 23)). This survey covers the entire FSMP clinical use process, including nutritional (risk) screening, nutritional status assessment, nutritional diagnosis, nutritional therapy, the preparation and distribution of FSMP preparations, the monitoring of ADRs, and FSMP charges.

### 2.3 Investigation method

The questionnaires were sent to 27 provinces/autonomous regions/municipalities (including Tianjin, Shanghai, Zhejiang, Shandong, Yunnan, Guangxi, Chongqing, Jiangsu, Beijing, Guangdong, Ningxia, Hunan, Sichuan, Guizhou, Hebei, Hubei, Xinjiang, Jilin, Fujian, Neimenggu, Heilongjiang, Shanxi, Anhui, Liaoning, Hainan, Henan, and Qinghai) through clinical nutrition quality control centers. Tertiary medical institutions that had established clinical nutrition departments and introduced FSMPs were eligible for inclusion. Four medical institutions were randomly selected for investigation from each province/autonomous region/municipality. The person in charge of the department of clinical nutrition of each institution was invited to complete the questionnaire.

### 2.4 Survey questionnaire quality control

Questionnaires that met one of the following criteria were considered invalid:

- Completion of less than 95% of the questions.
- Duplicate response by the same medical institution.
- Repeated test questions in the questionnaire had inconsistent answers.

### 2.5 Data analysis

A database was established using Excel 2016 software, and the data were analyzed using SPSS 19.0 statistical software. Qualitative data were described using frequency and the composition ratio (%). If quantitative data were normally distributed, the data were described using the mean and standard deviation. If not, the data were described using the median and interquartile range (IQR). For the frequency of ward rounds, once more than 3 days, once every 3 days, once every 2 days, once a day, and two times a day were assigned the values 1, 2, 3, 4, and 5, respectively. The Wilcoxon signed-rank test was used to compare the frequency of general ward rounds for hospitalized patients who have used FSMP after the first visit but had not yet reached the target dose with the frequency of routine ward rounds for hospitalized patients.

## 3 Results

### 3.1 Basic information

The distribution of 108 questionnaires was planned. Because FSMPs have not been used in Qinghai and Heilongjiang Provinces (the inclusion criteria were not met), 100 questionnaires were actually distributed, and 98 valid questionnaires were ultimately recovered for a recovery rate of 98%.

**Figure 1:**
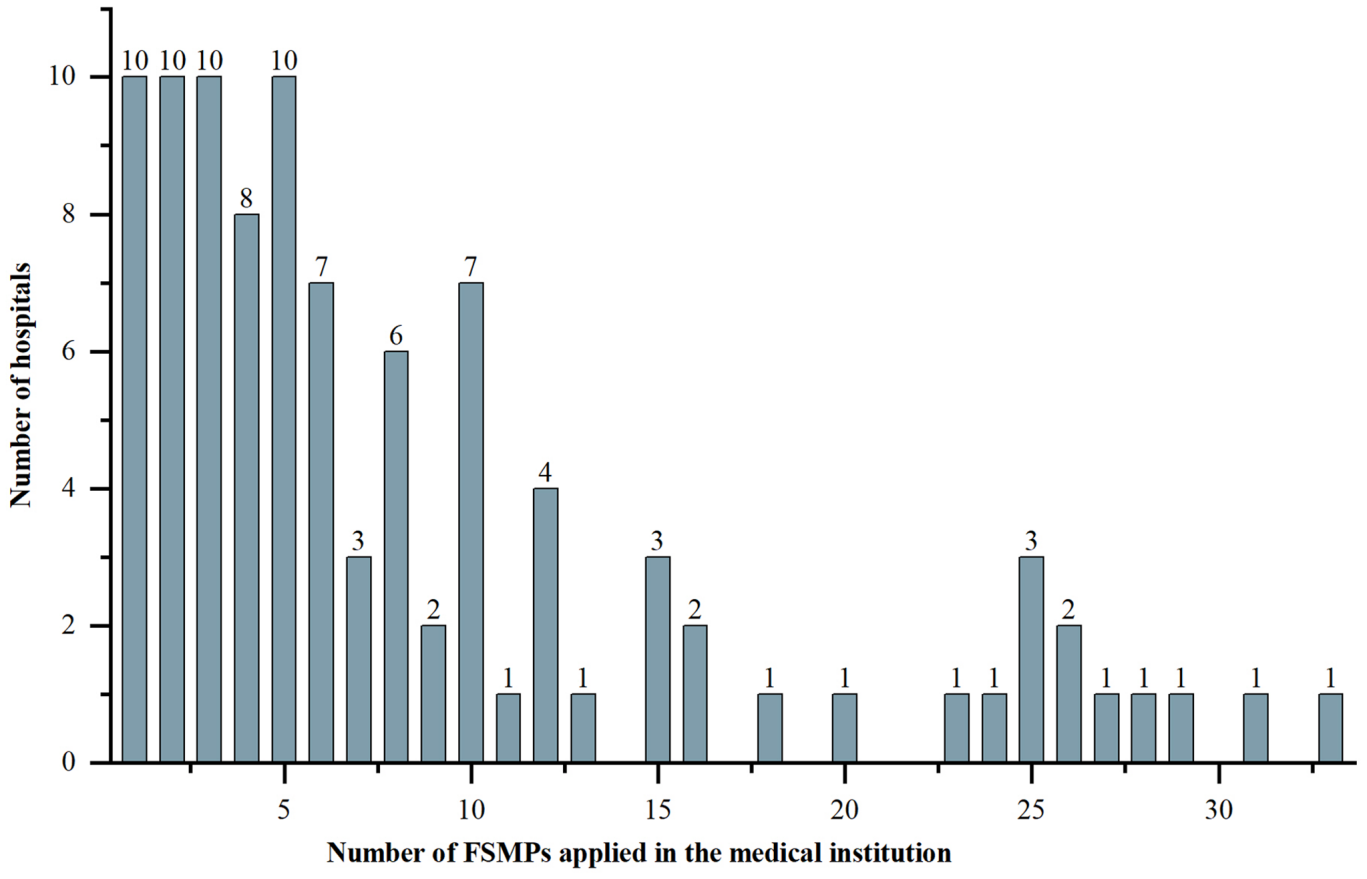
Distribution of the number of FSMPs applied in the medical institutions. FSMP, food for special medical purpose.

The majority of the surveyed hospitals were tertiary A hospitals (Table 1). The number of FSMPs used ranged from 1 to 33, with an IQR of 8.25 and a medium of 6. The detailed distribution is shown in Figure 1. The majority of hospitals had established enteral nutrition preparation rooms, in which 27 (30.68%) hospitals used laminar flow standards.

**Table 1:** Basic information on the medical institutions.

Basic information		*n* (%)
Hospital grade	Tertiary A	89 (90.82)
	Tertiary B	8 (8.16)
	Other	1 (1.02)
Enteral nutrition preparation room	Yes	88 (89.80)
	No	10 (10.20)
FSMP counter	Yes	18 (18.37)
	No	80 (81.63)
Charge code	Yes	45 (45.92)
	No	53 (54.08)

FSMP, food for special medical purpose.

### 3.2 Nutritional risk screening

**Figure 2:**
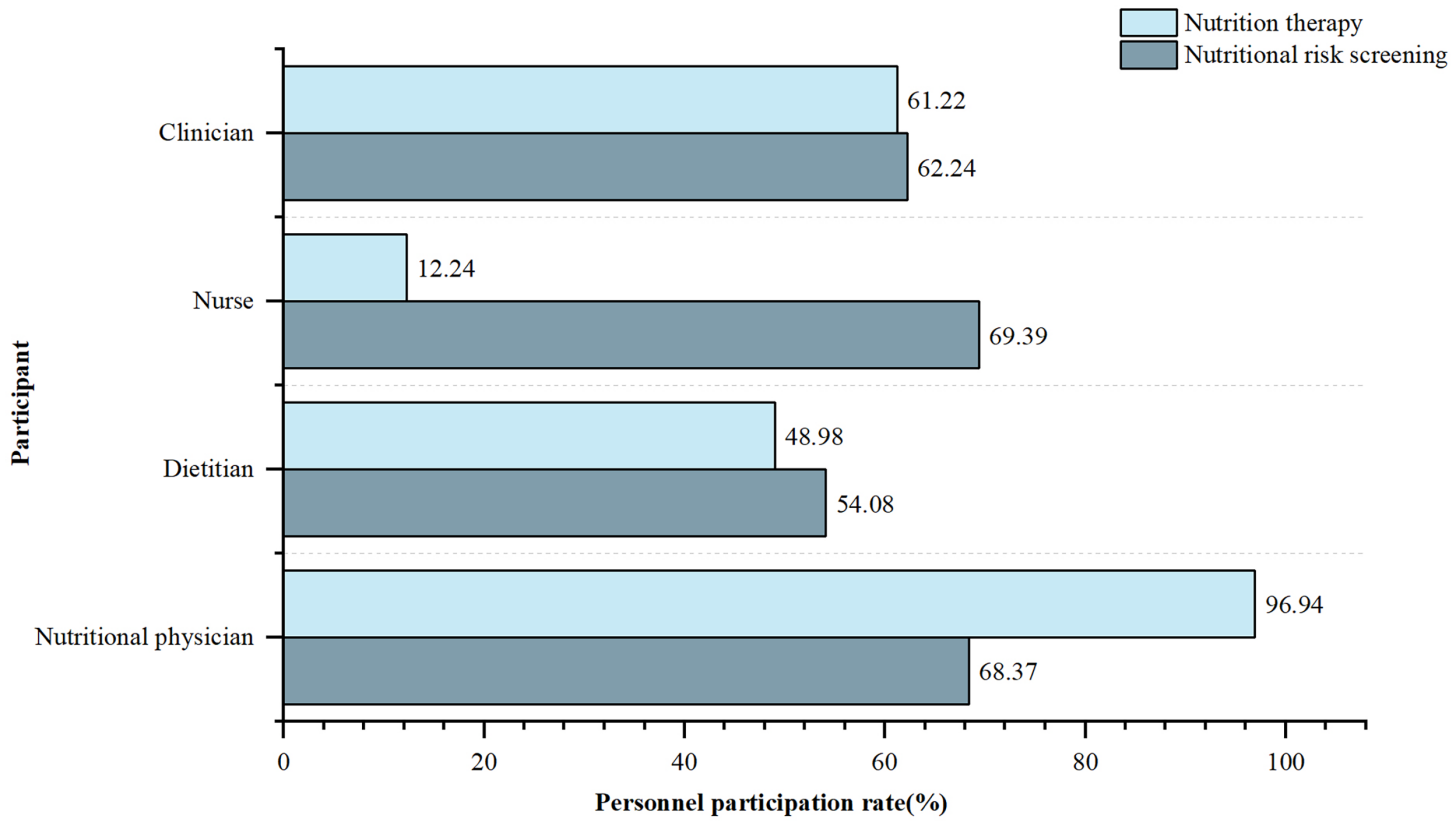
Participation of different types of personnel in nutritional risk screening and nutrition therapy. Personnel participation rate, Number of hospitals with the specific personnel participation in nutritional risk screening or nutritional therapy/total number of hospitals surveyed. Clinician, refers to physician who works in clinical departments (except for clinical nutrition departments). Nutritional physician, refers to physician who works in clinical nutrition department.

Ninety-two (93.88%) hospitals implemented active nutritional risk screening, while six (6.12%) implemented passive screening only after clinicians requested a consultation. Physicians working in the department of nutrition (nutritional physicians), dietitians, nurses and clinicians were all involved in implementing nutritional risk screening (Figure 2). It was conducted in 93 (94.90%) hospitals prior to prescribing FSMPs.

### 3.3 Nutritional assessment and diagnosis

Ninety-two (93.88%) hospitals conducted further nutritional assessments after nutritional risk screening. Nighty-six (97.96%) hospitals made nutritional diagnoses. Of them, 55 (57.29%) hospitals recorded the results on the front sheet of the medical records, and 94 (97.92%) recorded them in the consultation notes. The other hospitals recorded this information in the disease course, discharge summaries, nutrition assessment forms, and other places.

### 3.4 Nutritional therapy

Only 53 (54.08%) hospitals obtained written informed consent from patients before nutritional intervention. Eighty-eight (89.80%) hospitals had qualification requirements for those who prescribed FSMPs. Among them, nutritional physicians, dietitians, nurses, and clinicians accounted for 96.94, 48.98, 12.24, and 61.22%, respectively. These professionals in 69 (70.41%) hospitals received relevant training and passed the corresponding assessments before prescribing. Before prescribing, only 65 (66.33%) hospitals had an audit process conducted by superior clinicians or dietitians. Regarding FSMP prescription forms, 55 (56.12%) used only electronic prescriptions, 22 (22.45%) used only paper prescriptions, and the remaining 21 (21.43%) used both forms. Among them, 61 (62.24%) FSMP medical orders were embedded in the information system for information management.

### 3.5 Preparation and distribution of FSMP preparations

**Figure 3:**
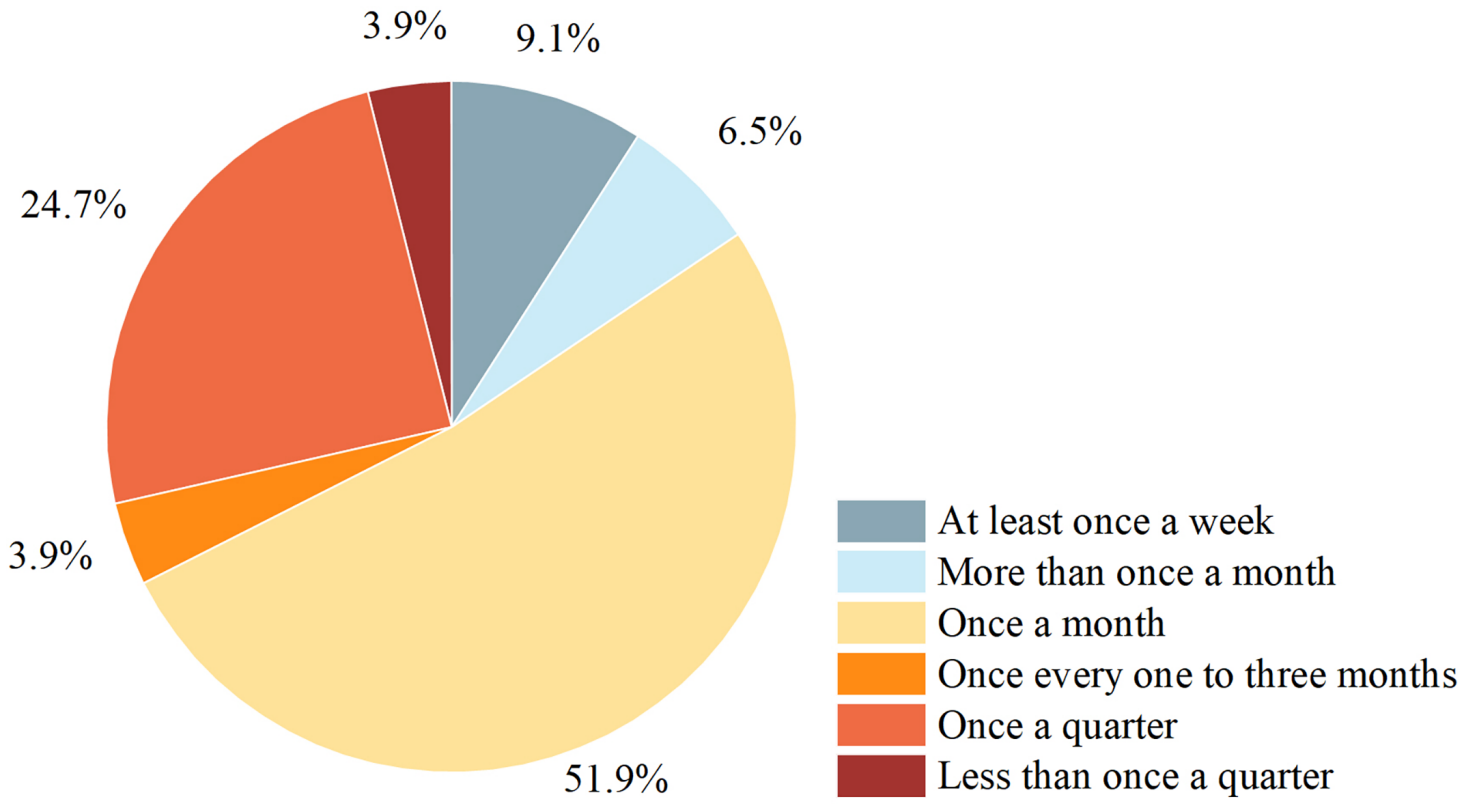
Frequency of nosocomial infection surveillance for the enteral nutrition preparation rooms. Nosocomial infection surveillance, quality testing of airborne bacterial concentrations, colony counts of dispensers’ hands, enteral nutrition solutions, operating tables and utensil colony counts.

Regarding air cleanliness, the enteral nutrition preparation rooms of nine (10.23%) hospitals had laminar flows of 300,000, 14 (15.91%) had 100,000, two (2.27%) had 10,000, two (2.27%) had 100, and the rest were non-laminar flow. The preparation and distribution of FSMPs in 77 (87.50%) hospitals were included in the hospital infection monitoring. Most hospital infection assessments were conducted once a month (accounting for 51.95%, Figure 3).

Physicians, dietitians, nurses, other staff, and pharmacists participated in FSMP preparation. FSMPs in 70 (71.43%) hospitals were distributed by full-time delivery staff from the department of clinical nutrition. FSMPs were delivered to the wards through intelligent transmission belts in six (6.12%) hospitals, by central transportation in three (3.01%), and by third-party companies in 16 (16.33%). Patients or their families were allowed to take their FSMPs in 33 (33.67%) hospitals. Before delivery, 95 (96.94%) hospitals had a verification mechanism. Checks included ensuring that the outer packaging bag was undamaged, verifying that the patient’s name, department, quantity, and kind of FSMP preparations matched the prescription, and ensuring that the preparation label was clear and comprehensive. After the FSMP preparations were delivered to the ward, the patients themselves or family members kept them in 36 (36.73%) hospitals; in 27 (27.55%) hospitals, they were kept by the nurses, and these two custodianship methods coexisted in 33 (33.67%) hospitals. In all hospitals without independent enteral nutrition preparation rooms, FSMP could be purchased in its original packaging by can or by bottle. In three hospitals, FSMP was also available for purchase through non-hospital sources (pharmacies and other networks).

Only 18 hospitals (18.37%) had set up FSMP counters for patients, half of which were clearly marked in the special sales area (or special counters) for FSMP. Outpatients from 79 (80.61%) hospitals received FSMP in the department of clinical nutrition.

### 3.6 Adverse reaction monitoring

**Figure 4:**
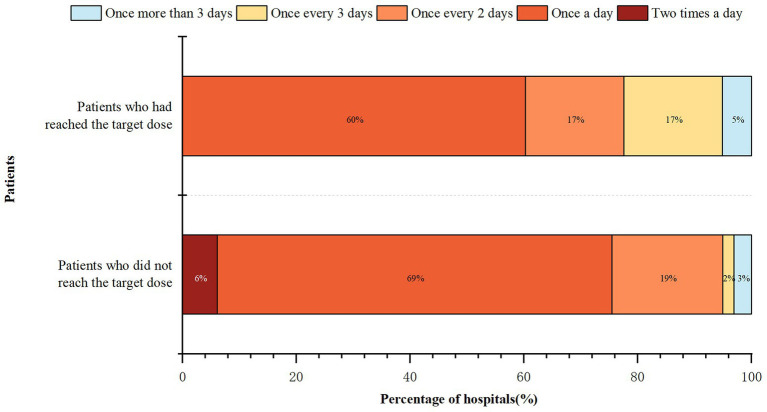
Frequency of ward rounds in the medical institutions.

The frequency of ward rounds in the medical institutions is shown in Figure 4. Hospitalized patients who had used FSMP after the first visit but had not reached the target dose received more frequent hospital rounds than patients who had achieved the target dose (*Z* = -4.372, *p* < 0.001). The frequency of routine ward rounds for more than half of the hospitalized inpatients was once a day. Post-discharge follow-up was implemented in 57 (58.16%) hospitals.

The proportions of hospitals that monitored stool conditions, gastrointestinal ADRs, sensory ADRs, metabolic complications and allergic reactions were 93.88, 96.94, 86.73, 90.82, and 90.82%, respectively. Sixty-four (65.31%) hospitals had established monitoring and treatment plans for FSMP ADRs. The ADR monitoring results were recorded by 82 (83.67%) hospitals, of which 46 (56.10%) were recorded electronically. The proportion of ADR monitoring data reported to the provincial or municipal clinical nutrition quality control center, FSMP management committee, and clinical management team of the department were 50.00, 25.61, and 79.27%, respectively.

### 3.7 FSMP charges

Detailed information on FSMP charges is shown in Table 2. Fewer than half of the hospitals had a charge code. Various charge names were used in hospitals without charge codes. In addition to meals, enteral preparations, and consumables, the following names were also used by medical institutions: FSMP, medical materials, treatment, nutrients, nutritional intervention, food, preparation, nutrition therapy for special diseases (a charging code in Hubei Province), medical service, enteral high nutrition treatment, self-paid drugs, product name, nutritional diet, select medicated diet on the basis of differential diagnosis, individualized nutrition guidance, and others.

**Table 2:** FSMP charges of the medical institutions.

Charging		Medical institutions *n* (%)
Charging approach	Unified charge	62 (63.27)
	Separate charge	25 (25.51)
	WeChat/Alipay third-party charge	7 (7.14)
	Other	4 (4.08)
Charge code	Yes	45 (45.92)
	No	53 (54.08)
Inpatient charge pathway	Outpatient system	35 (35.71)
	Inpatient system	63 (64.29)
FSMP items charged	Meal fee	33 (33.67)
	Enteral preparation fee	30 (30.61)
	Consumables	6 (6.12)
	Other	29 (29.59)

FSMP, food for special medical purpose.

### 3.8 Core link

**Figure 5:**
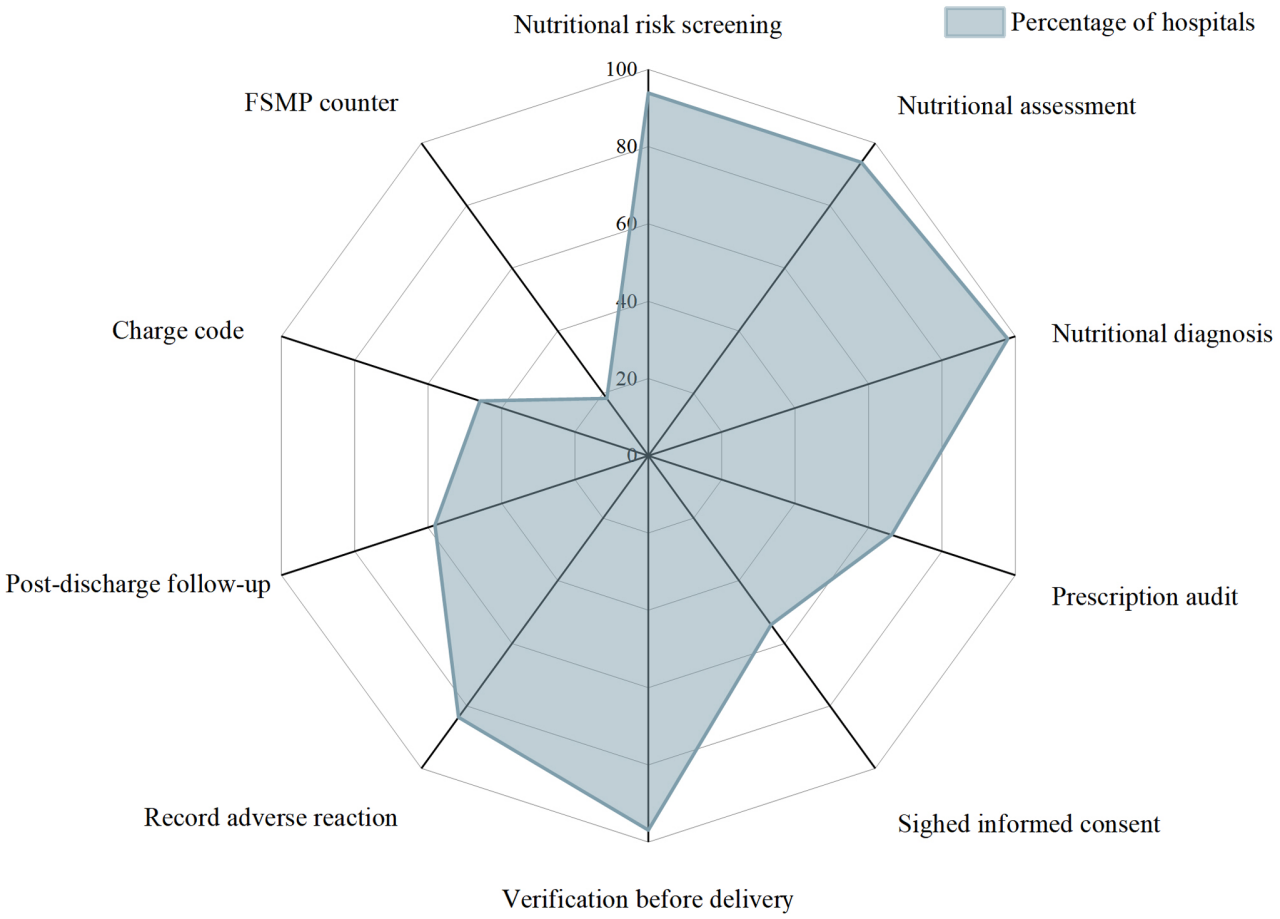
Status of core links during FSMP clinical use. FSMP, food for special medical purpose.

The execution rate of 10 FSMP clinical application core links or elements in the surveyed hospitals is shown in Figure 5.

## 4 Discussion

A number of studies have shown the effectiveness of FSMP in the nutritional treatment of disease (24–26). The use of FSMP in the European Union was shown to reduce one death for every 12 patients and medical costs by 12% per year. FSMP was introduced to China fairly recently, and a standardized clinical application pathway has not yet been established.2 However, China has introduced a number of supporting regulations and policies since the introduction of FSMP, including clinical trial requirements and clinical application standards, and has imposed strict regulations. We have limited access to the relevant regulatory approaches and experiences of other countries with the clinical application of FSMP, which may be due to different national conditions. However, we hope to introduce the Chinese model and current situation to the world.

This was the first survey to investigate the present condition of the FSMP clinical pathway in China. The stratified random sampling of 98 medical institutions in China showed that the proportion of FSMP introduced in the surveyed provinces/autonomous regions/municipalities directly under the central government was 92.60%. Only a few provinces reported that FSMP had not been introduced. These findings suggest that FSMP has been applied extensively at China’s tertiary medical facilities for nutritional therapy. In several provinces, the unsuccessful introduction of FSMP was mostly due to the failure to identify a reasonable charge pathway, which led to potential integrity risk concerns that could not meet the decision-making standards of the hospital’s directorate.

The “Expert Consensus on Clinical Management of FSMP (2021 Edition)” proposed that the application of FSMP should follow four standard processes: nutritional screening, assessment, diagnosis, and treatment (22). The survey results showed that the rates of active nutritional risk screening, nutritional status assessment, and diagnosis before FSMP therapy all reached more than 90%. These steps are also the steps recommended by the ASPEN guidelines for standardized nutritional support therapy (27), and have been well practiced in FSMP clinics. The process path of nutritional screening-assessment-diagnosis-treatment has been fully implemented.

However, this study also suggested diversity and the non-standardization of current clinical pathways. First, the number of FSMPs varied widely among the surveyed hospitals. This may be related to regional differences in procurement policies. For example, Jiangsu Province was the first province in China to include special medical food in the sunshine recruitment of medical insurance on the provincial public trading platform (28). As of June 2023, the number of hanging net products reached 79. Nevertheless, in more provinces, hospitals bid on their own, which may limit the number and scale of FSMPs. For dietitians, FSMP is a powerful weapon that can play a very good role in nutrition therapy. Over time, the imbalance of FSMP products will cause the uneven development of clinical applications in various provinces and cities in China. From the perspective of incorruptible risks, more attention needs to be paid to the transparency of access policies rather than limiting the number and types of products in hospitals. In fact, the number of registered enterprises and products in China continues to increase and has far surpassed overseas enterprises, occupying a dominant position. The government can coordinate the planning, similar to the centralized procurement in Jiangsu, and help hospitals to optimize the entry threshold of FSMP, which is conducive to the creation of a good healthcare environment and market environment.

The survey results showed that nutritional physicians, dietitians, nurses, and clinicians were all involved in nutritional risk screening. According to the “Guidelines for the Construction and Management of Clinical Nutrition Department” issued by the National Health Commission of the People’s Republic of China (29), nutritional risk screening should be completed by medical staff with relevant professional qualifications and training. Although the document does not explicitly identify specific personnel, it mentions that the primary physician is the first person responsible for nutritional risk screening. In addition to nutritional physicians, dietitians, and clinicians, nurses prescribed FSMP in 12.24% of the hospitals. However, they did not have the corresponding prescribing authority in China. According to the National Food Safety Standard General Rules for Food for Special Medical Purpose (GB 29922-2013) (1), FSMP should be used under the guidance of a physician or dietitians. This is similar to the situation in the US, where FSMP is required to be taken under the supervision of a physician or other qualified healthcare professional. However, in practice, federal law does not prohibit the dispensing of FSMPs without a prescription (5). Only 70.41% of the above personnel in healthcare institutions had undergone relevant training before prescribing FSMPs, and the training was conducted infrequently. In summary, there was an unclear division of labor and unclear qualifications of personnel related to FSMP clinical use. Thus, the division of labor should be clarified to improve the rationalization of clinical FSMP application. The first thing that needs to be clarified as well as firmly implemented is the FSMP prescribing authority, which is an important guarantee of the scientific validity of the FSMP therapy.

Guaranteeing the cleanliness of the FSMP preparation environment is a prerequisite for food safety. An unclean preparation environment and unstandardized preparation operation can easily lead to the contamination of enteral nutrition solutions, thus increasing the risk of hospital infections. Regular cleaning and hospital infection inspections of enteral nutrition preparation rooms are necessary. Recently, the Expert Consensus on Nosocomial Infection Management for Preparation of Enteral Nutrition Solution for Hospitalized Neonates recommended testing the effectiveness of air purification at least quarterly. Medical institutions have not yet standardized the frequency of hospital infection inspections, but about half of them choose to do it once a month.

In addition to the scientificity of FSMP prescription, it is especially important to dynamically adjust the treatment plan according to the patient’s condition and their feedback after consuming FSMP. Regular rounds can be conducted to monitor the latest condition of the patient, the fulfillment of the prescription and feedback according to the patient’s taste, as well as the presence of gastrointestinal intolerance. More frequent dietitian rounds are needed when increasing the dose and adjusting the formula, while the frequency of rounds can be slightly reduced during the maintenance phase after the therapeutic goals have been achieved. This reduction also represents the reality of the current shortage of clinical nutrition professionals in China.

The average length of stay continues to decrease under China’s medical insurance policy, leaving a shorter window for in-hospital treatment for clinical nutrition. Due to the limited length of hospital stay and illness factors, the prevalence of malnutrition at discharge after FSMP therapy remains high, possibly even higher than that at admission (30). Thus, follow-up for patients who require home enteral nutrition after discharge could significantly reduce medical expenses (31). Therefore, post-discharge follow-up and continued FSMP therapy are necessary. However, 41.84% of healthcare facilities did not provide post-discharge follow-up, suggesting that patients might purchase FSMPs through online shopping or in pharmacies without a prescription review or professional guidance, which might reduce their effects and have safety risks. Studies have shown that patients with oral nutritional supplements prescribed by a physician or dietitians have greater compliance than those without prescriptions (32). Considering the current shortage of nutrition professionals, low labor cost follow-up can be achieved with the help of AI telephone follow-up and records or internet platforms.

In China, FSMP is more strictly regulated in hospitals’ distribution chains than ordinary food products. The administrative measures for the operation and use of FSMP issued by medical institutions in multiple provinces require medical institutions to denote special areas or counters for FSMP, and it must not be mixed with ordinary food or drugs for sale (33–36). Signage shall be set up to indicate the words “special sales area (or counter) of food for special medical purposes.” Some of these provinces, including Shandong, Anhui and Jiangsu Provinces, require that the words be white on a green background and that the font be bold (33, 34, 36). The current proportion of FSMP counters is extremely low, and only 10% were clear labeling. Supervision by the General Administration of Market Supervision and Administration should be increased to promote the establishment of specific counters.

In the open-ended questions, several medical institutions mentioned doubts about the content of ADR monitoring. Uncertainty about the content and reporting criteria of ADR monitoring may lead to ineffective reporting or no reporting, which is not conducive to the implementation and transparency of the reporting mechanism. With reference to drugs, we suggest that an FSMP ADR should be defined as an uncomfortable or harmful reaction due to the use of a qualified FSMP that occurs under normal usage and dosage, is unrelated to the purpose of the medication, and usually resolves after the discontinuation of the FSMP. ADRs should be distinguished from complications, such as infectious complications. However, adverse metabolic complications arising from the use of FSMP should be considered. The “Management Measures for FSMP Adverse Reaction Monitoring in Sichuan Province” was written by experts organized by the Sichuan Provincial Market Supervision Administration in 2018, but no similar documents have been released by other provinces in China. Unifying the content of ADR monitoring and reporting is conducive to FSMP post-market supervision and public safety safeguarding.

Unified coding and charging have long been problems in clinical nutrition in China. Each province’s “FSMP Clinical Application Management Standard” demands unified coding and charging (37–39), which would help to promote the standardized clinical application of FSMP. The results showed that 45.92% of the surveyed hospitals had achieved unified coded charging for FSMP, which was only slightly higher than the result in a 2022 (43.3%) survey by our research team (7). More than 20 different types of charges were reported, mainly for meals and enteral preparations, similar to the 2022 survey. The lack of reasonable charge types will, firstly, prevent FSMP from entering hospitals through smooth bidding and, secondly, reduce patients’ willingness to buy them. The regulatory authorities need to clarify the classification of FSMP and determine its special attributes, which are different from those of ordinary foodstuffs, drugs or medical consumables. In the short term, it can be combined with the hospital diet system to standardize charges and application paths, and in the long term, it is necessary to formulate accurate coding and financial categorization.

Also, whether a particular FSMP is covered by basic health insurance or commercial insurance has a huge impact on patient costs for accessing the FSMP. Researchers compared FSMP reimbursement methods in 14 countries and found that China was the only one that did not cover it at all (40). Reimbursement methods differ greatly across countries, even in the US, private health insurance coverage for FSMP is not uniform across all insurers or states (5). More effort is required to standardize FSMP charges. The introduction of coordinated and rationalized reimbursement policies for nationally covered from public or private health care providers would gone a long way towards increasing FSMP practices (41).

**Figure 6:**
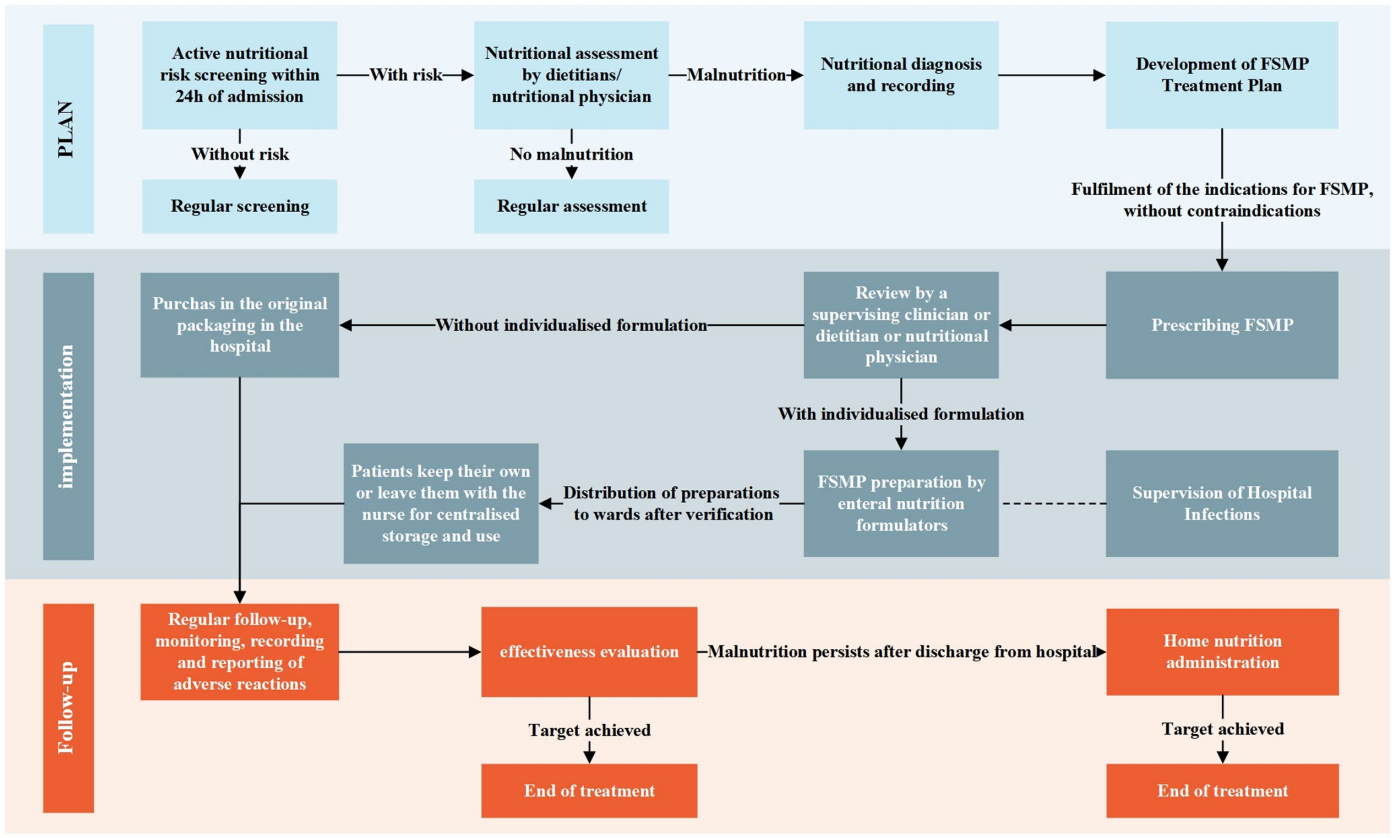
The standardized clinical pathway for FSMP in China.

We constructed a standardized clinical pathway for FSMP in China to solve the above problems (Figure 6). The main body of the path is divided into two lines depending on whether it is individualized or not. Based on our findings, the standardized application of FSMP in Chinese clinical practice can be further promoted in the future through standardized pathways, improving regulatory policies, and introducing international experiences so that more patients can receive high-quality nutritional support.

We welcome nutrition workers from other countries to share their clinical FSMP application pathways with us. China imposes stringent regulatory requirements on FSMPs and sets a high entry threshold at the primary stage, which is conducive to standardized FSMP management and also provides a good market environment for the orderly development of such food products. Compared with developed countries, China needs to further strengthen its basic research capability and the implementation of FSMP regulations and standards, strengthen the construction of legal normative systems, conduct research and evaluate clinical application, and continue to improve FSMP regulations in China.

There were some shortcomings in this study. The sample size of this study was limited, stratified sampling did not take into account the organization, financing, and number of hospital Beds of the medical institutions, and the sampled medical institutions were mainly tertiary A hospitals. Therefore, the survey results might be biased. A comparative analysis of the current status of pathways in different levels of hospitals was not performed. The questionnaire was distributed through clinical nutrition quality control centers; however, quality control centers have not been established nationwide, so very few provinces, cities, and autonomous regions were not included. Thus, the status of the FSMP clinical pathway was probably overestimated.

## 5 Conclusion

An FSMP clinical pathway prototype (Nutritional Screening-Assessment-Diagnosis-Treatment) has been implemented in China’s tertiary medical institutions While opportunities exist, many issues remain to be addressed.: Imbalance of FSMPs among hospitals, unknown qualifications of personnel and division of labor, inadequate prescription review and rounds, lack of post-discharge follow-up and charge code. The application of FSMP in Chinese clinical practice should be further promoted through standardized pathways, strengthening teamwork, improving regulatory and reimbursement policies, and introducing international experiences to maximize the value of FSMP nutritional therapy.

## Data Availability

The raw data supporting the conclusions of this article will be made available by the authors, without undue reservation.
