# Supplementary material for: Towards a clinical pathway to food for special medical purpose in China: current progress based on a cross-sectional survey
Source: Front Med (Lausanne). 2025 Jul 4;12:1538105. doi: 10.3389/fmed.2025.1538105 (PMC12271200; doi:10.3389/fmed.2025.1538105)
Supplement: Supplementary file 1 [file Table_1.docx]

**Investigation of the current status of the clinical use pathway of food for special medical purpose (FSMP) in medical institutions**

1. Name of your hospital: [fill in the blank]

_________________________________

2. Province or city: [fill in the blanks]

_________________________________

3. Rank of your hospital: [choose one]

| ○ Tertiary A |
| --- |
| ○ Tertiary B |
| ○ Other _________________ |

4. How many FSMPs are currently used in your hospital? (number) [fill in the blank]

_________________________________

5. What is the form of nutritional (risk) screening? [choose one]

| ○ Active screening |
| --- |
| ○ Passive screening (screening after consultation with the doctor) |
| ○ Active screening + passive screening |

6. Which of the following personnel in your hospital carry out nutritional (risk) screening? [multiple choice]

| □ Nutritional physician |
| --- |
| □ Dietitian |
| □ Nurse |
| □ Clinician |
| □ Other _________________ |

7. Was a nutritional (risk) screening conducted before an FSMP prescription was issued? [choose one]

| ○ Yes |
| --- |
| ○ No |

8. Is further nutritional status assessment performed on patients after nutritional (risk) screening? [choose one]

| ○ Yes |
| --- |
| ○ No (please jump to question 11) |

9. Are nutritional status assessment results embedded in your hospital HIS system in the form of electronic documents? [choose one]

| ○ Yes |
| --- |
| ○ No |

10. Is nutritional diagnosis performed? [choose one]

| ○ yes |
| --- |
| ○ No (please jump to question 13) |

11. Where is the location of the nutritional diagnosis record? [multiple choice]

| □ The first page of the medical record |
| --- |
| □ Consultation note |
| □ Other _________________ |

12. Before nutritional intervention, is the corresponding informed consent signed by the patient? [choose one]

| ○ Yes |
| --- |
| ○ No |

13. Are the indications and contraindications checked before formulating a nutritional treatment plan and prescribing FSMP? [choose one]

| ○ Yes |
| --- |
| ○ No |

14. What are the above indications? [multiple choice]

| □ Difficulty in eating orally, insufficient feeding, or inability to eat orally |
| --- |
| □ Patients with gastrointestinal diseases with poor digestion or absorption |
| □ Perioperative nutritional support and accelerated surgical rehabilitation |
| □ Adjuvant treatment for tumor chemotherapy and radiotherapy |
| □ Patients with a high metabolic state |
| □ Patients with organ dysfunction |
| □ Other _________________ |

15. What are the above contraindications? [multiple choice]

| □ Acute severe pancreatitis |
| --- |
| □ Severe stress, paralytic ileus, upper gastrointestinal bleeding (mild mucosal bleeding can be treated with enteral nutrition) |
| □ In patients with complete intestinal obstruction, gastrointestinal peristalsis is severely slowed, severe abdominal distension affects respiratory function, and the amount of gastric retention exceeds 400–500 mL/d |
| □ Small bowel resection within 4–6 weeks |
| □ Patients who are prone to developing dumping syndrome after subtotal gastrectomy |

16. Does your hospital require the person who issues the FSMP prescription and instructs the patient to use it to have the corresponding qualifications? [choose one]

| ○ Yes |
| --- |
| ○ No |

17. Which personnel in your hospital are qualified to issue FSMP prescriptions and guide patients to use them? [multiple choice]

| □ Nutritional physician |
| --- |
| □ Dietitian |
| □ Nurse |
| □ Clinician |
| □ Other _________________ |

18. Are the above personnel qualified to prescribe FSMPs and guide patients to use them after training and passing the examination? [choose one]

| ○ Yes |
| --- |
| ○ No |

19. Are prescriptions reviewed by a senior clinical physician or a nutritional physician/dietitian before they are issued? [choose one]

| ○ Yes |
| --- |
| ○ No |

20. What is the FSMP prescription form used in your hospital? [choose one]

| ○ Electronic prescribing |
| --- |
| ○ Handwritten paper prescription |
| ○ Electronic prescribing + handwritten paper prescriptions |

21. Does your hospital have an FSMP preparation room? [choose one]

| ○ Yes, laminar flow level, level _________________ |
| --- |
| ○ Yes, not laminar |
| ○ No (please jump to question 25) |

22. Is the preparation and distribution of formulas in the preparation room included in hospital infection control monitoring? [choose one]

| ○ Yes |
| --- |
| ○ No (please jump to question 25) |

23. What is the frequency of hospital infection examinations? [fill in the blank]

_________________________________

24. Who prepares FSMP in your hospital? [multiple choice]

| □ Dietitian |
| --- |
| □ Nutritional physician |
| □ Nurse |
| □ Service personnel |
| □ Pharmacist |
| □ Other _________________ |

25. What is the form of preparation distribution? [multiple choice]

| □ Department of clinical nutrition distribution |
| --- |
| □ The intelligent transmission belt delivers to the ward |
| □ Central transportation |
| □ Direct delivery by a third party |
| □ Patient self-provision |
| □ Other _________________ |

26. Do the distributing personnel check with the preparing personnel before the formulation is distributed? [choose one]

| ○ Yes (please jump to question 28) |
| --- |
| ○ No |

27. What does the above check include? [multiple choice]

| □ Whether the outer packaging bag is intact |
| --- |
| □ Whether the number, type, and prescription of the FSMP preparations are consistent |
| □ Whether the patient's name and department are consistent with the prescription |
| □ Whether the preparation label is complete and clear |
| □ Other _________________ |

28. What information is included in the FSMP preparation label? [multiple choice]

| □ Inpatient department |
| --- |
| □ Bed number |
| □ Admission number |
| □ Preparation date |
| □ Quality guarantee period |
| □ Condition of storage |
| □ Contact number for the preparation room or related production organization |
| □ Main nutrient components (e.g., energy, protein, fat, and carbohydrates) |
| □ Warning (e.g., no intravenous injection) |
| □ Other _________________ |

29. If there is no independent preparation room in the hospital, what is the FSMP access route? [multiple choice]

| □ Purchase in original packaging in the hospital (prescription read by nutritionist) |
| --- |
| □ Purchase outside the hospital (pharmacy or Internet) |
| □ Other _________________ |

Depends on question 21, option 3

30. Is there a special FSMP cabinet for patients in the hospital? [choose one]

| ○ Yes |
| --- |
| ○ No (please jump to question 34) |

31. Is there a clear sign in the special medical use formula food sales area (or counter) for this special section? [choose one]

| ○ Yes, the specific words are_________________ |
| --- |
| ○ No |

32. What information does your hospital check before the patient goes to the special cabinet to collect FSMP? [multiple choice]

| □ Prescription information (name, prescription quantity, etc.) |
| --- |
| □ Payment information |
| □ Other _________________ |

33. What is the storage method of FSMP preparations after they are delivered to the ward? [choose one]

| ○ Dispersed storage (family members or patients retain it) |
| --- |
| ○ Centralized storage (to be placed and stored by nurses) |
| ○ Dispersed + centralized storage |
| ○ Other _________________ |

34. What is the storage time of FSMP bags for hospitalized patients? [fill in the blank]

_________________________________

35. How to access FSMP for outpatients? [choose one]

| ○ Special cabinets for collection in this court |
| --- |
| ○ Department of clinical nutrition |
| ○ Out-of-hospital approaches |
| ○ Other _________________ |

36. For hospitalized patients who have used FSMP after the first visit but have not yet reached the target dose, what is the frequency of general ward rounds? [choose one]

| ○ Once per day |
| --- |
| ○ Twice per day |
| ○ Once every two days |
| ○ Other _________________ |

37. What is the frequency of routine ward rounds for hospitalized patients? [choose one]

| ○ Once every day |
| --- |
| ○ Once every two days |
| ○ Once every three days |
| ○ Other _________________ |

38. Under what circumstances will the use of (possible reuse) FSMP be suspended for inpatients in your hospital? [multiple choice]

| □ Digestive tract hemorrhage |
| --- |
| □ Disturbance of carbohydrate metabolism |
| □ Fasting before surgery |
| □ Postoperative gastrointestinal paralysis |
| □ Gastrointestinal dysfunction or certain conditions that require gastrointestinal rest |
| □ Failure to follow nutritional advice |
| □ Other _________________ |

39. Under what circumstances will the use of FSMP be discontinued for inpatients in your hospital? [multiple choice]

| □ Patient refuses |
| --- |
| □ Family members refuse |
| □ The attending physician requested discontinuation |
| □ Continued use of FSMP is no longer yielding additional benefits |
| □ Return to normal eating |
| □ Other _________________ |

40. After discharge, do hospitalized patients receive follow-up? [choose one]

| ○ Yes |
| --- |
| ○ No |

41. Is the clinical application (prescription) of FSMP embedded in the information system of the medical institution for information management? [choose one]

| ○ Yes |
| --- |
| ○ No |

42. What adverse reactions to FSMP are monitored? [multiple choice]

| □ Stool condition (e.g., constipation) |
| --- |
| □ Gastrointestinal adverse reactions (e.g., acid reflux and bloating) |
| □ Adverse sensory reactions (e.g., taste) |
| □ Metabolic complications (e.g., electrolyte metabolic abnormalities) |
| □ Allergic reactions (e.g., skin allergic reactions) |
| □ Other _________________ |

43. Does your hospital have a plan for monitoring and handling adverse reactions to FSMP? [choose one]

| ○ Yes |
| --- |
| ○ No |

44. Are the adverse reaction monitoring results recorded? [choose one]

| ○ Yes |
| --- |
| ○ No (please jump to question 48) |

45. In what form are adverse reaction monitoring results recorded? [choose one]

| ○ Paper records |
| --- |
| ○ Electronic records |

46. To which institutions will your hospital report adverse reaction monitoring data? [multiple choice]

| □ Provincial or municipal clinical nutrition quality control center |
| --- |
| □ FSMP Management Committee |
| □ Department clinical management team |
| □ Other _________________ |

47. What is the charging method for FSMPs in your hospital? [choose one]

| ○ Integrated and unified in the hospital system |
| --- |
| ○ Charged separately |
| ○ WeChat/Alipay third-party charging |
| ○ Other _________________ |

48. Is there a charge code? [choose one]

| ○ Yes |
| --- |
| ○ No |

49. Who are the people charged if charges were separately done? [choose one]

| ○ Third-party |
| --- |
| ○ Hospital finance |
| ○ Other _________________ |

Depends on question 47, option 2

50. What is the charging path for hospitalized patients? [choose one]

| ○ Section for outpatients |
| --- |
| ○ Hospital system |

51. What are your hospital’s FSMP charges? [choose one]

| ○ Meal expenses |
| --- |
| ○ Cost of enteral preparation |
| ○ Consumables |
| ○ Other _________________ |
